# ALK-Positive Squamous Cell Carcinoma Dramatically Responded to Alectinib

**DOI:** 10.1155/2018/4172721

**Published:** 2018-03-18

**Authors:** Ray Sagawa, Takehiko Ohba, Eisaku Ito, Susumu Isogai

**Affiliations:** ^1^Department of Pulmonology, Ome Municipal General Hospital, Ome, Japan; ^2^Department of Pathology, Ome Municipal General Hospital, Ome, Japan

## Abstract

Anaplastic lymphoma kinase (ALK) rearrangement is usually observed in patients with adenocarcinoma. Herein, we report a case of squamous cell carcinoma (SCC) with ALK rearrangement treated with alectinib. The patient was a 73-year-old woman without a smoking history. She consulted us with nonproductive cough and loss of appetite. Computed tomography scan revealed a mass in the left lower lobe of the lung. According to the pathological examinations, we diagnosed the tumor as SCC. Because the patient had never smoked, we searched for driver mutations and found that the tumor harbored ALK rearrangement. We began treatment with alectinib, and the tumor remarkably reduced in volume. No severe adverse events were observed. Although there are only few reports of SCC with ALK rearrangement, this case implies that clinicians should consider searching for driver mutations in patients with SCC when there are atypical findings or characteristics.

## 1. Introduction

Several oncogenic driver mutations, such as those of the anaplastic lymphoma kinase (ALK) genes, are treatable with molecular targeting drugs. ALK rearrangement is usually observed in patients with adenocarcinoma at a frequency of approximately 4%, and it is thought to be rarely expressed in squamous cell carcinoma (SCC) [1, 2].

Herein, we present a case of chemotherapy-naïve SCC with ALK rearrangement successfully treated with alectinib.

## 2. Case Presentation

**Figure 1:**
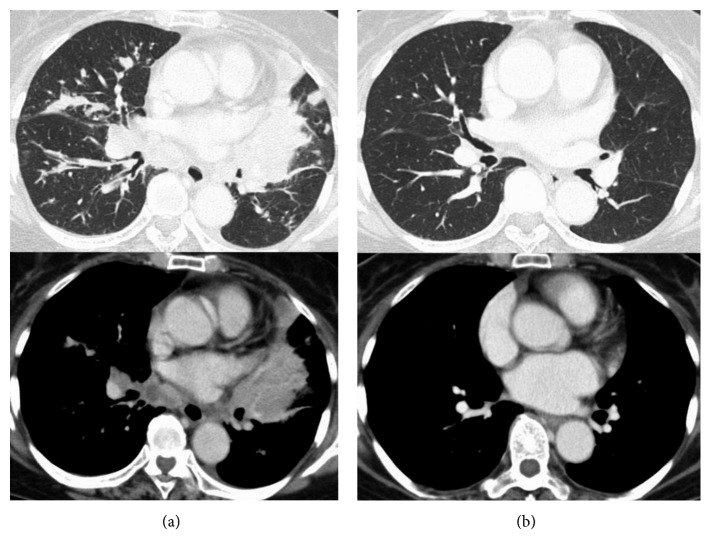
CT findings before and after the treatment. (a) Chest CT at admission. It showed a tumor in the left lower lobe of the lung with mediastinal lymphadenopathy, pleural dissemination, and multiple pulmonary metastases in both of the lungs. (b) CT revealed dramatic tumor reduction in the primary and metastatic lesions, and this had maintained after 9 months since we started the treatment.

**Figure 2:**
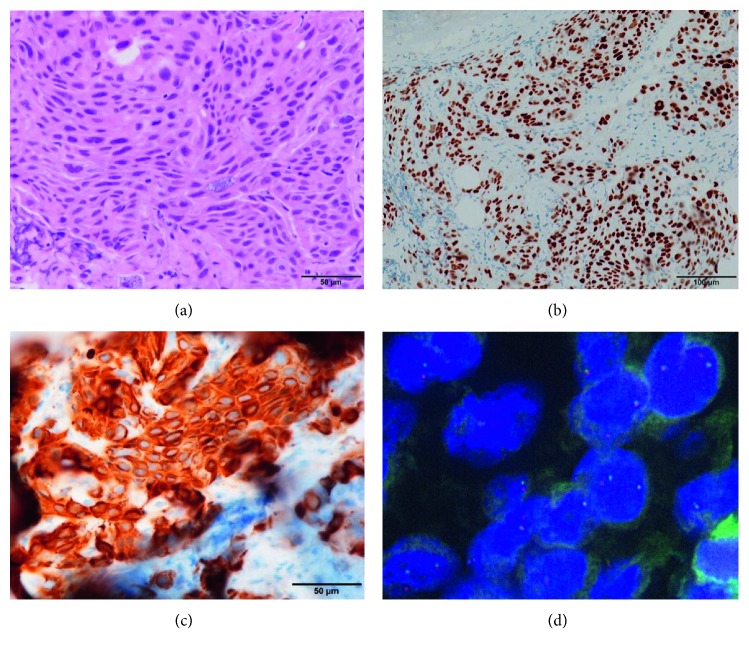
Pathological examinations. (a) Hematoxylin and eosin staining showed moderately differentiated malignant cells with abundant cytoplasm connected with intercellular bridge. Immunohistochemical staining revealed that the tumor cells were strongly positive for p40 (b) and CK5/6 (c). (d) Fluorescence in situ immune-hybridization signals of ALK. Split red and green signals can be observed.

A 73-year-old woman with no smoking history presented to our hospital with nonproductive cough and loss of appetite. Chest computed tomography (CT) scan showed a mass with a diameter of 38 mm in the left lower lobe of the lung. The patient also had mediastinal lymphadenopathy, pleural dissemination, and multiple pulmonary metastases throughout both lungs (Figure 1(a)). Pathological examination of the transbronchial biopsy specimen revealed cancer with solid growth. Hematoxylin-eosin staining showed cytoplasm-abundant tumor cells connected by intercellular bridges. Keratinization was not clear (Figure 2(a)). Some Alcian blue-positive mucus was also observed. Immunohistochemistry (IHC) showed that the tumor cells were diffusely and strongly positive for p40 and cytokeratin (CK) 5/6 (Figures 2(b) and 2(c)) but completely negative for thyroid transcription factor-1 (TTF-1) and napsin A. Although few cells (less than 5% of all) showed adenomatous differentiation, most of them had typical characteristics of SCC. Based on these findings, we diagnosed the tumor as moderately differentiated SCC. Despite the diagnosis of SCC, the patient underwent ALK testing, as she had no history of smoking. IHC analysis revealed the tumor cells were diffusely and strongly positive for the ALK antibody. Fluorescence in situ hybridization confirmed the presence of ALK gene rearrangement with a rearrangement-positive cell rate of 95% and higher (Figure 2(d)).

The clinical stage was T4N3M1b (PLE, PUL, and OSS), and we began to treat the patient with alectinib as the first-line therapy, according to the guideline. Coughs and anorexia had dramatically improved in a few days, and we observed that the primary lesion shrunk remarkably in a week. After 9 months, a follow-up CT scan revealed remarkable size reductions in the primary lesion (38 mm to 7 mm, to be specific) and regression of mediastinal lymphadenopathy, and multiple pulmonary metastases had been maintained (Figure 1(b)), which we diagnosed as good partial response, according to the RECIST criteria. Occasional grade 1 leukopenia was the only adverse effect observed.

## 3. Discussion

We presented a rare case of SCC with ALK rearrangement, which showed a remarkably positive response to alectinib. IHC analysis revealed that TTF-1 and napsin A were completely negative, whereas p40 and CK5/6 were diffusely and strongly positive. These IHC findings strongly suggested that the tumor was SCC. Morphologically, most tumor cells had typical characteristics of SCC. There was a slight possibility that the tumor was not pure SCC; however, analysis of the acquired tissue specimens confirmed the diagnosis of SCC.

Clinicians do not necessarily examine SCC tumors for ALK rearrangement owing to the rarity of such cases [1]. However, the present case had no smoking history, which is atypical in a patient with SCC. Therefore, we attempted to examine driver mutations and detected ALK rearrangement in the tumor. This case highlights the importance of searching for driver mutations in patients with SCC when there are atypical findings or backgrounds.

The efficacy of ALK-targeted therapies for treating ALK-positive SCC is controversial. To the best of our knowledge, there are only two reports of ALK-positive patients with SCC treated with alectinib. Tamiya et al. reported a case wherein the patient had a heavy smoking history and did not respond well to alectinib [3]. However, Mamesaya et al. reported an ALK-positive patient with SCC without smoking history who was successfully treated with alectinib [4], which was similar to our case. These cases implied that smoking status may be a predicting factor for alectinib treatment in ALK-positive patients with SCC. However, further case reports are required to confirm such an association.

In conclusion, we have reported a rare case of SCC with ALK rearrangement that showed a remarkably positive response to alectinib. We should thus consider searching for driver mutations in patients with SCC when there are atypical findings or characteristics in order to examine other treatment choices in some cases.
